# Morphological Design and Synthesis of Nanoparticles (Second Edition)

**DOI:** 10.3390/nano16040255

**Published:** 2026-02-15

**Authors:** Mirela Honciuc, Andrei Honciuc

**Affiliations:** “Petru Poni” Institute of Macromolecular Chemistry, Gr. Ghica Voda Alley 41A, 700487 Iasi, Romania

Nanoparticles exhibit size- and shape-dependent properties that differ from those of bulk materials, which make them suitable for a variety of applications in catalysis, biomedicine, energy storage, sensing, and environmental remediation. In these contexts, morphology—including particle size, shape, anisotropy, and internal structure—has been shown to significantly influence performance and application potential. Morphological control is therefore not just a structural consideration but a functional requirement in nanoparticle design.

At the same time, the synthetic method used to produce nanoparticles determines not only their morphology but also their chemical composition, surface functionality, and scalability. Recent efforts have focused on improving synthesis protocols in two main directions: (i) by developing green chemical approaches that minimize environmental impact, such as using plant-derived reducing agents or renewable precursors, and (ii) by implementing engineering strategies such as flow-based systems, microfluidics, and process modeling to achieve reproducibility, precision, and scalability.

This second edition of the Special Issue “Morphological Design and Synthesis of Nanoparticles” presents contributions that address both morphology and synthesis as critical and interconnected factors in determining nanoparticle function. The selected articles cover experimental, theoretical, and modeling approaches, with an emphasis on structure–property relationships, sustainable synthesis, and processing techniques that enable application-specific control of nanoparticle characteristics.

The nine selected contributions are organized into four thematic categories that reflect current research directions in nanoparticle science:

(i) Green and engineered approaches to nanoparticle synthesis and morphological control, highlighting sustainable chemical methods and flow-based engineering strategies for achieving precise nanoparticle morphologies;

(ii) Asymmetry, interfacial assembly, and functional behavior of Janus nanostructures, focusing on how surface anisotropy and interface-driven self-assembly impact nanoparticle functionality;

(iii) Size-dependent electronic and magnetic properties of nanoparticles, addressing how variations in particle dimensions affect band structure, electronic transitions, and multifunctional behavior; and

(iv) Nanoparticle–matrix interactions and their effect on collective material behavior, examining how nanoparticles modulate the structural and dynamic properties of the surrounding material environment, particularly in soft matter systems.

*Green and Engineered Approaches to Nanoparticle Synthesis and Morphological Control:* Green chemistry synthetic strategies represent a pathway to a sustainable future. The article authored by Wasilewska et al. [1] presents a sustainable approach to the synthesis of silver nanoparticles by employing apple extract as a natural reducing agent. This study explores the influence of synthesis parameters, particularly pH, on the size, shape, and distribution of the resulting nanoparticles, demonstrating that tuning these conditions enables control over morphology. It is important to note that the Ag nanoparticles generated this way, with sizes ranging between 6 and 30 nm, did not form tight aggregates because they were efficiently stabilized against aggregation by active compounds in the apple extract. This green chemistry strategy exemplifies an environmentally friendly and benign alternative to conventional chemical routes, utilizing the phytochemical constituents of the extract to facilitate the reduction of silver ions. The Ag nanoparticles with apple extracts as reducing agents demonstrated promising antimicrobial properties, with high activity against Gram-positive bacteria and fungus. This work not only highlights the importance of natural product-based nanoparticle synthesis but also underscores the ability of phytochemicals to also stabilize nanoparticle colloids against aggregation and provide surface functionalities for targeted uses.

Girard et al. [2] also follow green chemistry principles to create environmentally friendly routes to nanomaterial synthesis. The authors present a comprehensive study on the modeling and optimization of lignin nanoparticle synthesis via anti-solvent precipitation—a method valued for its simplicity and scalability. Lignin is an aromatic biopolymer consisting of three main interconnected monomeric units: p-hydroxyphenyl, guaiacyl, and syringyl. Among multiple uses, lignin can be used to produce bio-composites, biodegradable plastics, carbon fibers, and adhesives as a sustainable alternative to petroleum-based materials and can be used in energy and fuel, electronics and batteries, nanoparticles and drug delivery, etc. While raw lignin mass can be obtained via different chemical methods, such as the Kraft method by treating wood with sodium hydroxide and sodium sulfide at high temperature or through “organosolv” processes, the latest advances have focused on obtaining lignin as nanoparticles for added material value. Lignin exhibits enhanced functionality in nanoparticle form, such as improved antibacterial, anti-oxidant, and UV protection properties, among others, greatly expanding its application potential. By developing a predictive framework based on experimental data, Girard et al. [2] correlate process parameters such as solvent/anti-solvent ratio, lignin concentration, and stirring rate with resulting nanoparticle size and distribution. The work enables precise control over morphological features, which is essential for tailoring the physicochemical properties of lignin nanoparticles for applications in cosmetics, drug delivery, and sustainable products. This study not only advances our understanding of lignin-based nanomaterials but also emphasizes the critical role of process modeling and process engineering in achieving desired nanoparticle morphologies through green and renewable sources.

The research article by Wong et al. [3] presents a novel approach to producing prednisolone nanoparticles using a Dean instability-based microfluidics mixer. This work broadens the engineering toolkit for bottom–up nanoparticle fabrication, offering a scalable, energy-efficient, and reaction-free strategy relevant to drug formulation, nanomedicine, and advanced materials design. This hydrodynamic technique exploits secondary flow patterns in curved microchannels to achieve rapid mixing and controlled nanoprecipitation, resulting in nanoparticles with diameters as small as 46 nm. The study systematically investigates how flow rate, solvent composition, and channel geometry influence particle morphology and size distribution. By enabling continuous, scalable synthesis under mild conditions, this method offers a promising alternative to conventional batch techniques, particularly for pharmaceutical applications where precise control over drug particle size is critical in bioavailability and therapeutic efficacy. This work highlights the interplay between fluid dynamics and nanoparticle morphology, which could be of significance in advanced drug delivery system development.

*Asymmetry, Interfacial Assembly, and Functional Behavior of Janus Nanostructures*: A second group of papers addresses some fundamental and practical aspects of asymmetric Janus nanoparticles. For example, Nidhi et al. [4] challenge the traditional assumption that explicit Janus morphology is a prerequisite for achieving unidirectional mobility in nanoscale systems. Through a combination of experimental synthesis and motion tracking experiments, the authors demonstrate that spontaneous symmetry breaking can occur even in gold-coated nanoparticles lacking clear biphasic structures. When gold is isotropically deposited onto silica nanoparticles, the resulting particles exhibit sufficiently asymmetric features to induce autonomous motion under catalytic decomposition of hydrogen peroxide, similarly to their Janus counterparts. This work not only expands the conceptual framework of what constitutes a “mobile” nanoparticle but also suggests that simpler and more scalable fabrication routes can yield functional nanomotors.

The article by Honciuc et al. [5] introduces an innovative method for fabricating two-dimensional nanostructured films by interfacing Langmuir–Blodgett and Pickering emulsion techniques. This hybrid approach enables the assembly of silica nanoparticles at the oil–water interface in a Pickering emulsion. The Pickering emulsion droplets formed are spread onto the air–water interface of a Langmuir–Blodgett trough and then compressed into a compact monolayer and polymerized. Due to a preferential distribution of nanoparticles between oil/water and air/oil interfaces, thin-film asymmetric Janus monolayers of highly ordered nanoparticles are obtained. The resulting nanostructured Janus films exhibit tunable morphology and enhanced functionality, which could be particularly useful in photonic applications. The presence of ligand functionality on the surface of nanoparticles imparts these films with heavy metal ion adsorption capability; for example, copper ions can be captured from aqueous solutions. This work demonstrates how interfacial self-assembly and morphological control at the nanoscale can be synergistically employed to design advanced nanostructured materials.

Furthermore, the article authored by Honciuc M. and Honciuc A. [6] provides a comprehensive review of amphiphilic Janus nanoparticles (JNPs), emphasizing their role as scalable analogs to molecular surfactants in interfacial science. The review outlines how the intrinsic anisotropy of JNPs—defined by distinct hydrophilic and hydrophobic surface regions—enables them to adsorb and partition at liquid–liquid and liquid–air interfaces, reduce surface tension, and self-assemble into complex suprastructures such as vesicles, monolayers, and foams. The authors explore the physicochemical principles governing these behaviors, including interfacial forces, immersion depth, and environmental responsiveness. By drawing parallels to molecular amphiphiles and highlighting unique advantages such as structural rigidity and tunable geometry, the article establishes JNPs as versatile building blocks for applications ranging from emulsion stabilization to active nanomotors and responsive materials.

*Size-Dependent Electronic and Magnetic Properties of Nanoparticles*: A notable contribution in this issue is the study by Shilov et al. [7], which examines how the electronic band gap of tantalum-based nanoclusters depends on particle size. Using magnetron sputtering combined with quadrupole mass filtering, the authors synthesized monodisperse Ta and Ta_2_O_5_ nanoclusters (1.5–8 nm) with controlled oxidation during deposition. XPS and REELS analyses showed that for both core–shell (Ta/TaOx) and fully oxidized Ta_2_O_5_ nanoclusters, the band gap increases markedly as the size drops below 6 nm—demonstrating clear quantum confinement effects. Band gap values reached up to 7.55 eV, significantly higher than the bulk value, underlining the influence of nanoscale dimensions on material properties. The authors also linked these changes to the effective electron mass, providing a theoretical framework for band structure tuning. This work highlights how particle size can be used as a design parameter to engineer wide-band-gap nanomaterials for applications in optical coatings, nonlinear optics, and UV photodetectors.

The work authored by Apostolov et al. [8] highlights the potential of microscopic models and Green function theory to model the magnetic and electronic properties of ferroelectric potassium niobate (KNbO_3_) nanoparticles. The authors explore how co-doping KNbO_3_ nanoparticles with combinations of rare-earth and transition metal ions can enhance their multifunctional properties. Using theoretical modeling, the authors show that co-doping with ions such as La/Cr or La/Co results in nanoparticles that exhibit both ferroelectricity and ferromagnetism at room temperature, along with a significantly reduced band gap. This multiferroic behavior is tunable through the choice and concentration of dopants, making co-doped KNbO_3_ nanoparticles promising candidates for future applications in multifunctional electronic and solar energy conversion devices.

*Nanoparticle–Matrix Interactions and their Effect on Collective Material Behavior:* The article authored by Drozd-Rzoska et al. [9] investigates the influence of BaTiO_3_ nanoparticles on the dielectric properties of the classic nematogenic liquid crystal (LC) 4-methoxybenzylidene-4′–butylaniline (MBBA) by changing the local morphology and interactions of the LC matrix. The authors reveal that even small additions of nanoparticles can significantly amplify so-called pretransitional effects—subtle molecular fluctuations that occur before a material undergoes a phase transition. Remarkably, these effects are detected not only in the liquid and nematic phases, but also in the solid state, where such behavior is rarely observed. This suggests that nanoparticles introduce localized interactions that alter the dynamics of phase change at the nanoscale. The work highlights how dielectric spectroscopy can serve as a sensitive probe of molecular organization and demonstrates the potential of nanoparticle additives to tune the phase behavior of soft matter systems in both fundamental and applied contexts.

Collectively, the contributions in this Special Issue emphasize that nanoparticle morphology is not merely a structural attribute but a key parameter that governs functional performance across diverse application domains. The works presented herein demonstrate how advances in synthetic methodologies—ranging from green chemistry to flow-based engineering—enable precise control over size, shape, and surface characteristics. Several studies highlight the critical role of morphology in modulating interfacial phenomena, including self-assembly, wettability, and selective adsorption. Others show how nanoparticle size influences bulk electronic and magnetic properties through confinement effects and how nanoscale additives impact the behavior of surrounding matrices, particularly in soft matter systems.
